# A Micro Bridge-Wing-Thickened Low-Energy Exploding Foil Initiator Chip

**DOI:** 10.3390/mi15050589

**Published:** 2024-04-28

**Authors:** Pengfei Xue, Heng Hu, Tao Wang, Peng Xiong, Mingyu Li, Qingxuan Zeng

**Affiliations:** 1 State Key Laboratory of Explosion Science and Technology, School of Mechatronical Engineering, Beijing Institute of Technology, Beijing 100081, China; xuepengfei95@126.com (P.X.); pxiongcn@163.com (P.X.); 2 China Ship Development and Design Center, Wuhan 430064, China; bitwangtao@163.com

**Keywords:** exploding foil initiator, energy utilization, electrical explosion, electrothermal simulation

## Abstract

To enhance the energy efficiency of exploding foil initiator systems (EFIs) and mitigate energy loss due to ablation in the bridge-wing regions, a low-energy bridge-wing-thickened EFI chip was designed and fabricated. Computational analysis revealed that increasing the thickness of the bridge flanks significantly reduces ablation within the bridge region during the electrical explosion. The refinement of the design led to the adoption of a bridge flank thickness of 19 μm, with the bridge area dimensions specified as 0.25 mm × 0.25 mm × 4 μm. This bridge-wing-thickened EFI chip was produced by employing micro-electro-mechanical systems (MEMS) technology and underwent rigorous performance evaluations. The empirical results closely matched the computational predictions, thereby corroborating the precision of the proposed model in simulating the temperature distribution seen during the explosion process. Notably, this enhanced EFI design achieves a flyer velocity of 3800 m/s at a condition of 900 V/0.22 μF, signifying a significant advancement in EFI system efficiency and performance.

## 1. Introduction

EFIs are inline detonation or ignition devices that originated from the idea of adding a layer of flyer and a hollow cylindrical accelerating chamber to the bridge membrane proposed by J.R. Stroud [1,2]. This system distinguishes itself from traditional initiation devices by its non-contact nature with respect to the explosive, requiring precise conditions of pulsed high current and voltage to actuate, thus ensuring enhanced safety and reliability measures [3,4,5,6,7,8]. Consequently, its application has proliferated across various ignition and detonation systems in military technology.

To enhance the safety profile of EFIs, the selection of energetic materials is strategically conservative. Since the advent of EFIs, there has been stringent governance over the choice of these materials. Specifically, hexanitrostilbene-IV (HNS-IV) has been sanctioned as the standard initiating explosive for detonation mechanisms, while boron potassium nitrate (BPN) has gained approval as a stable ignition compound for pyrotechnic initiation systems [9]. Research efforts and the ensuing analyses have consistently engaged with these two materials, underscoring their central role in the safety and reliability of EFI applications.

The process of EFI electrical explosion was studied by researchers at an early stage. During the electrical explosion process of the metal, the temperature at the four corners of the metal bridge foil rises initially. Subsequently, heat spreads along the outer edges of the bridge, suggesting that the bridge area undergoes uneven heating prior to the explosion, a phenomenon commonly referred to as the “hot spot effect” [10,11]. This thermal unevenness results in ablation around the periphery of the bridge, known as the bridge-wing area, leading to electrical energy dissipation. Therefore, the key to improving the energy utilization of EFIs is to make the energy primarily available for work in the bridge region, while reducing the loss of energy to other regions of the bridge-wing, thereby affecting the precision and effectiveness of the cutting and propulsion phases. Since the inception of EFIs, researchers have been exploring ways to improve its energy utilization.

The performance of EFIs is closely linked to the specifications of the metallic bridge foil, including its thickness and overall dimensions, which directly influence the electrical explosion process and subsequent energy conversion. Numerous studies [12,13,14,15,16,17] have demonstrated that there is an optimal match between the dimensions of the bridge region and the exploding foil’s susceptibility. When the thickness of the bridge foil is thicker, the higher the energy that is required for the conversion of the metallic state to the ionic state, and the lesser the energy used to drive the flyer, resulting in a lower flyer velocity. Conversely, when the bridge foil is excessively thin, it lacks the capacity to generate adequate expansive force to accelerate the flyer to its necessary threshold velocity.

Optimizing the structure of the bridge zone is another significant factor that can enhance energy utilization in EFIs. The shape of the bridge zone can markedly influence the effectiveness of the electrical explosion process. Circular-shaped bridge zones minimize the edge ablation seen in traditional square bridge zones, which leads to better energy conservation [18,19,20]. Additionally, the angle between the bridge zone and the surrounding wing structures plays a pivotal role. An angle of 45° has been shown to maximize burst power and energy utilization, outperforming other configurations [21]. Furthermore, transitioning from a single-strip bridge to a parallel array of bridges enhances energy utilization. Compared to square bridge foils, arrayed bridge foils have a smaller mass and greater resistance, which better matches the discharge characteristics necessary for efficient ignition. However, precision in manufacturing is critical, as any deviation between the designed and actual array bridge foils can result in synchronization issues during the electrical explosion process [22,23,24,25].

The effectiveness of metal electrical explosion devices also depends on the micro-morphology and the quality of the deposited film. A more uniformly distributed metal film that adheres more robustly to the substrate typically promotes better electrical explosion performance and necessitates lower energy to initiate the explosion. The film-forming quality of the foils used in exploded foil metal bridges mainly depends on the processing techniques used, which mainly include magnetron sputtering coatings for micro-electro-mechanical system (MEMS) technology, gap screen printing film-forming for low-temperature co-fired ceramic (LTCC) processes, and electroplating film-forming for printed circuit board (PCB) processes [26,27,28,29,30]. Of these, magnetron sputtering stands out as yielding the highest quality of films with great purity, superior density, and minimal surface roughness, usually around 10 nm.

The primary focus of the above study was on the influence of bridge foil dimensions, structural design, and manufacturing techniques on electrical explosion performance. However, it did not adequately address the issue of energy loss due to the ablation of the bridge-wing area during the metal electrical explosion process. Accordingly, the presented paper proposes an innovative design for EFIs in the form of a bridge-wing-thickened configuration. This design seeks to optimize energy efficiency within the EFIs by thickening the bridge-wing region to lower its resistance. Such a modification is intended to decrease the current density across the bridge-wing during the electrical explosive process, thereby focusing energy deposition primarily in the bridge region. This paper details the computational thermal field analysis made of the temperature distribution within thickened bridge-wing explosion foil. The test EFI was prepared utilizing MEMS technology. The prepared EFIs were then characterized regarding their properties.

## 2. Modeling of the Bridge-Wing Thickened EFI

**Figure 1:**
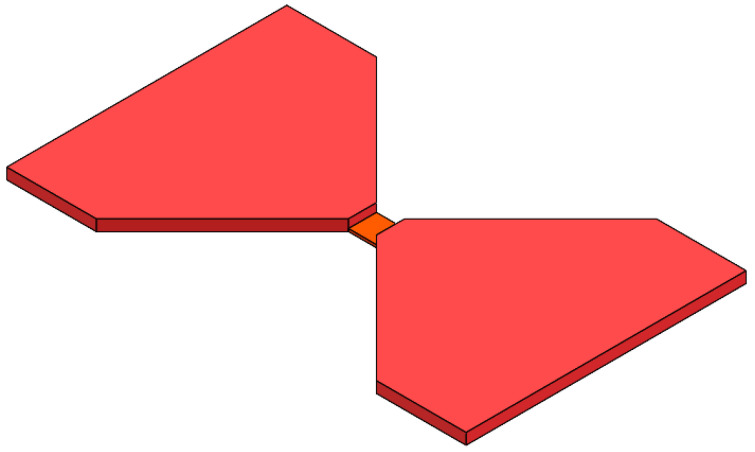
The structure of the bridge-wing thickened EFI.

Figure 1 illustrates the bridge-wing-thickened EFI chip, showing a structure with all the metal layers thickened except for the bridge region. The parameters of the EFI chip’s bridge area and the flyer’s thickness significantly influence the device’s performance output. In this study, the parameters are defined as referenced in the extant research [16,17]—a bridge area with a length and width of 0.25 mm and a height of 4 μm, and a flyer with a thickness of 15 μm. To circumvent any potential interference from the bridge-wings’ thickening on the flyer motion, the wings’ thickness is maintained at 19 μm, equal to that of the flyer, ensuring consistent and smooth kinetics.

To determine whether the proposed bridge-wing thickened EFI chip can alleviate the excessive ablation of the bridge-wing prior to the onset of electrical explosion (at 3000K), the study utilized a comprehensive multi-physics simulation platform to simulate and analyze the electro-thermal dynamics of the bridge foil during the electrical explosion process. The modeling process is as follows.

The bridge region is subject to the transient heat conduction equation [29] during the occurrence of a metallic electrical explosion:

(1) $$C_{\nu }(T)\rho \frac{dT}{dt}=\sigma (T){\left|E\right|}^{2}+\nabla \cdot \left(\lambda \nabla T\right),$$

where *C_v_* is the constant volume heat capacity of the metal bridge foil, J/kg/K; *ρ* is the density of the metal, kg/m^3^; *T* is the temperature, K; *t* is the time, s; *σ* is the metal conductivity, S/m; *E* is the electric field strength, V/m; and *λ* is the thermal conductivity, W/m/K.

In the metal electrical explosion process, the constant capacity heat capacity and metal conductivity, as well as thermal conductivity, are not constants but are instead temperature-dependent variables. The resistivity model, as well as the constant volume heat capacity model, in the metal electrical explosion process, are further optimized in our laboratory and can be expressed using the following equations [29,31,32]:

(2) $$C_{V,\mathrm{Cu}}\left(T\right)=\left\{\begin{array}{l} 0.377+1.055\times 10^{-4}\left(T-250\right)\;250\leq T<1356\\ 0.678-6.280\times 10^{-5}T\;1356\leq T<8000 \end{array}\right.,$$

(3) $$\sigma =\frac{10^{8}}{1.25+0.00675(T-250)}\;T\leq 3000,$$

(4) $$\lambda \left(T\right)=\left\{\begin{array}{l} -0.0685T+420.75\;250\leq T<1356\\ -0.02717T+364.54\;1356\leq T<8000 \end{array}\right..$$

When performing electrothermal simulation calculations, it is sufficient to take the parameters associated with the corresponding temperature. In addition, the calculation process must follow the current continuity equation:

(5) ∇⋅J=E⋅∇σ+σ⋅∇E=0,

where *J* is the metal bridge foil current density, A/m^2^.

The initial current in the model is calculated using the following equation:

(6) $$i_{\left(t\right)}=\frac{U_{0}}{\omega L}e^{-\delta t}\sin\omega t,$$

(7) $$\omega =\sqrt{\frac{1}{LC}-\frac{R^{2}}{4L^{2}}},$$

(8) $$\delta =\frac{R}{2L}.$$

where *i*_(*t*)_ is the initial current, A; *U*_0_ is the operating voltage, V; *R* is the loop resistance, mΩ; and *L* is the loop inductance, nH.

The metallic bridge foil of EFIs typically possesses a thickness that is measured at just a few microns, which is significantly less than the skin effect depth; this permits the exclusion of skin effect considerations from the computational analysis. Furthermore, due to the rapidity of the electrical explosion process in the metal foil, it is practical to neglect the thermal exchange between the bridge wire and its surroundings during this brief but intense event.

**Figure 2:**
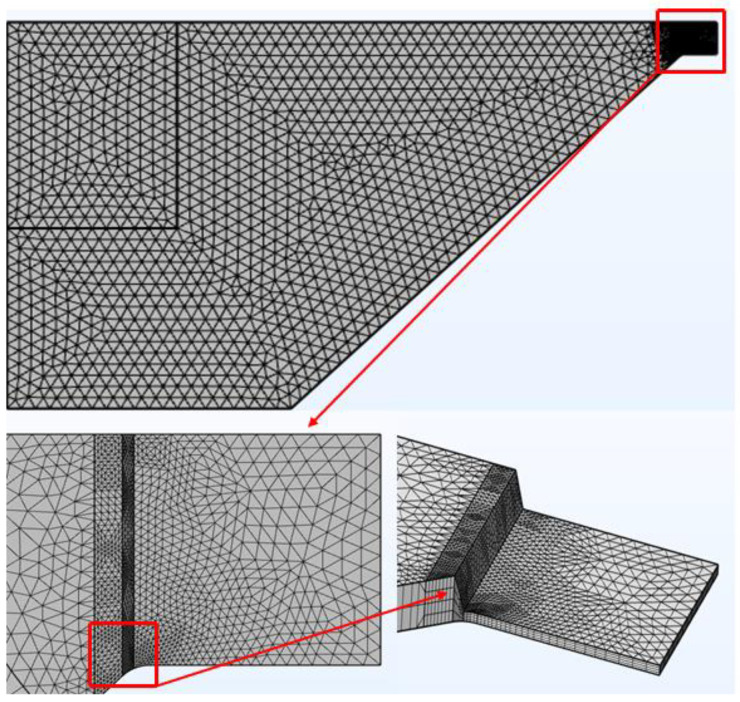
Mesh of the bridge-wing thickened model.

In formulating the computational model for the thickened-bridge-wing type of EFI, a geometric construct was established, followed by mesh discretization. Given that the metal bridge zone exhibits symmetrical architecture, computational efficiency can be optimized by constructing a quarter geometric model, effectively reducing the calculation load. The mesh division process is illustrated in Figure 2. The red markers in the Figure 2 are localized enlargements of key areas of thickening in the bridge area.

**Figure 3:**
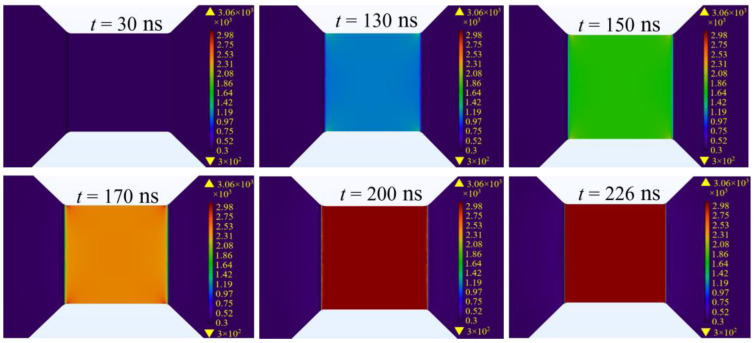
Electro-thermal simulation results of the bridge-wing thickened bridge foil.

For the simulation, the thickness of the bridge foil was specified as 4 μm, with a square bridge area having a side length of 0.25 mm. The initial charge voltage was set at 1000 V. The initial current profile was ascertained using Equation (6) from the discharge circuit, which accounts for a loop inductance of 102 nH and a resistance of 168.7 mΩ. The electro-thermal simulation results of the electrical explosion process in the metal bridge zone of the EFI are elucidated in Figure 3. Initially, the temperature of the bridge foil undergoes a gradual increase, with the bridge area sustaining the ambient temperature until 30 ns after energization. Up to 130 ns, the corners of the bridge foil exhibit a slow temperature rise, reaching 1356 K—the melting point—whereas the center of the bridge area achieves a temperature of approximately 900 K. Over the next 40 ns, the temperatures at the corners accelerate to 2800 K, with the central bridge area approaching 2500 K. This trend demonstrates a decreasing temperature gradient between the corners and the center of the bridge area as the duration of energization extends. By the 226 ns mark, all regions of the metal bridge attain the vaporization threshold of 3000 K. Furthermore, an examination of the temperature distribution on the sides of the bridge area, as depicted in Figure 3, reveals that no ablation occurred within any bridge-wing regions throughout the energization process until vaporization was reached. This uniform temperature distribution in the bridge area suggests that the bridge foil’s surface is uniformly capable of electrical vaporization simultaneously.

**Figure 4:**
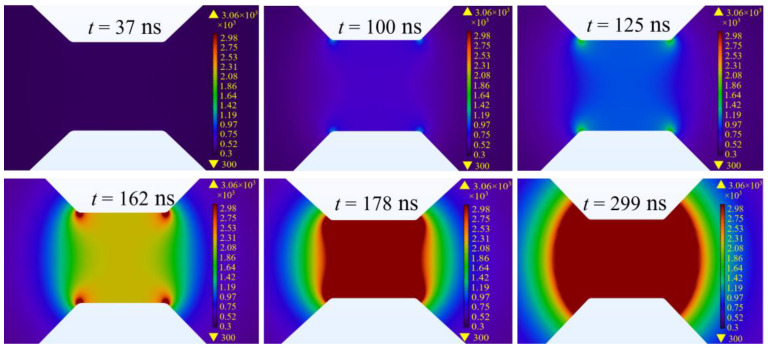
Electro-thermal simulation results for conventional square bridge foil.

Modeling the temperature field distribution for a traditional square bridge foil provides insights into its thermal dynamics under energization. Figure 4 illustrates that the initial significant temperature increase occurs at the corners of the square bridge after 100 ns of energization, reaching 1000 K, while the central region attains approximately 700 K. By 125 ns, the temperature at the center climbs to 1000 K, indicating a delayed thermal response relative to the corners. Continuing the energization up to 125 ns results in the corners heating up to 1600 K, with the center lagging at 900 K, highlighting an expanding thermal gradient between the corners and the center. As the current application persists, the corner temperatures surpass the vaporization threshold at 162 ns, with the central area reaching 2200 K, amplifying the temperature disparity. Subsequently, the temperature in the central region continues to rise gradually, gradually reducing the thermal gradient with the corners. By the 178 ns mark, almost all parts of the bridge achieve vaporization temperature, leading to the initiation of heating in the bridge-wings, which then experience a temperature increase. Once the metal bridge maintains a uniform temperature at the vaporization point, ablation begins in certain areas of the bridge-wings. This ablation process not only entails vaporization but also contributes to propagating the effect outward. By 299 ns, a considerable portion of the bridge-wing has undergone ablation, evidencing the dynamic progression of thermal and material responses throughout the EFI operation.

Compared with the temperature distribution results of the bridge-wing thickened EFI to those of the conventional square bridge, it is observable that during electrical explosion events, the conventional square bridge induces ablation in the bridge-wing area. Conversely, the thickened bridge-wing bridge avoids ablation during the electrical explosion process, concentrating almost all the energy into the bridge area itself. This is advantageous for enhancing the structural integrity and smoothness of the ejected flyer.

**Figure 5:**
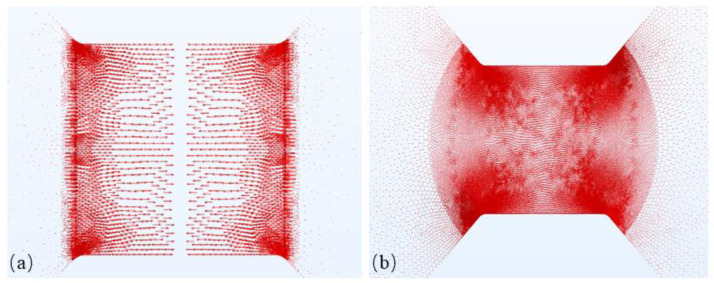
(**a**) Current density simulation result for bridge-wing thickened bridge foil. (**b**) Current density simulation result for conventional square bridge foil.

The current density in the bridge during the electrical explosion of metal has a crucial impact on the driving process of the flyer. When analyzing the current density distribution calculation results of the bridge shown in Figure 5, it is evident that the current density distribution pattern is broadly in line with the temperature distribution in the bridge area. The current in the metal bridge of the thickened bridge-wing EFI is primarily concentrated in the bridge area, with significantly lower current density in the bridge-wing transition region. Conversely, the current density in the corners of the conventional square bridge area is the highest and is also more densely distributed in the bridge-wing transition area.

## 3. Preparation of a Bridge-Wing Thickened EFI and Testing of Its Characteristics

### 3.1. Fabrication of the Bridge-Wing Thickened EFI

**Figure 6:**
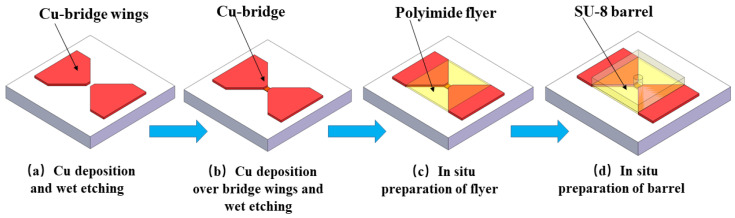
Fabrication process of the bridge-wing thickened EFI chip.

The bridge-wing thickened EFI chip was fabricated on a glass substrate using MEMS technology, as depicted in Figure 6:

(1) A copper film with a thickness of 15 μm was deposited onto the substrate using magnetron sputtering. Subsequently, a bridge-wing region featuring a square void with a length of 0.25 mm was fashioned via photolithography and etching.
(2) An additional 4 µm copper layer was sputtered onto a substrate that was already patterned with the bridge-wing area. A wet etching process was then employed to pattern the bridge area within the square void, resulting in a metal bridge foil characterized by a bridge-wing thickened design.
(3) A polyimide photoresist was spin-coated on top of the metal bridge foil, which was then subjected to photolithography to pattern the layer directly above the metal bridge area, yielding a final thickness of 15 µm.
(4) Finally, a barrel of approximately 300 μm was fabricated in situ over the flyer layer, using SU-8 photoresist, to complete the microstructure.

**Figure 7:**
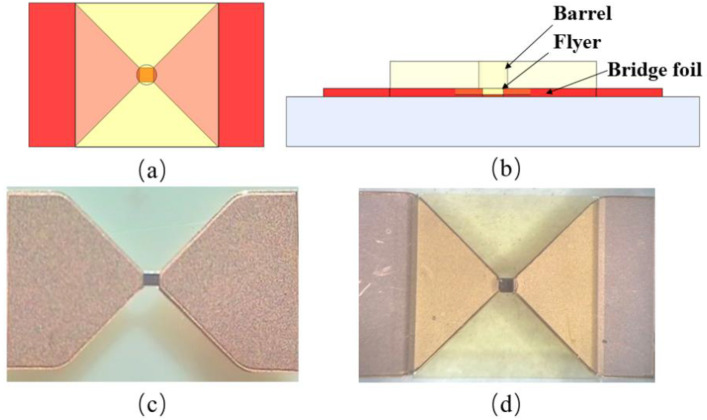
Structure schematic and physical drawing of a bridge-wing thickened EFI chip. (**a**) Top view of EFI. (**b**) Front view of EFI. (**c**) Bridge-wing thickened bridge foil. (**d**) Bridge-wing thickened EFI chip.

The schematic diagram, as well as the physical figure of the bridge-wing thickened EFI chip, are demonstrated in Figure 7. It can be seen that an EFI chip prepared using MEMS technology has a complete and standardized structure and meets the design requirements.

### 3.2. Electrical Explosion Performance Test

**Figure 8:**
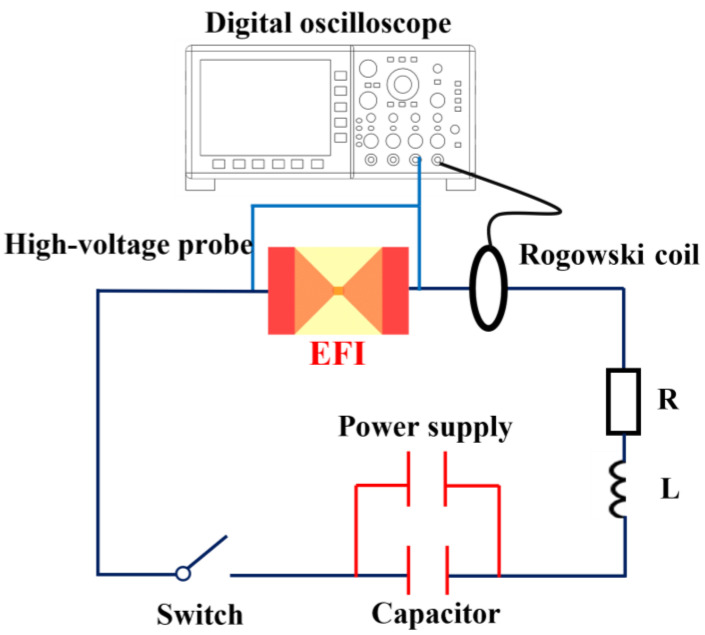
Circuits for EFI electrical explosion performance characterization.

In order to study the electrical explosion performance of the bridge-wing thickened EFI chip, the test rig was set up as shown in Figure 8. The test rig includes a discharge circuit, a Rogowski coil for current measurement, a high-voltage probe, and a digital oscilloscope. The discharge circuit is configured with a high-voltage power source, a capacitor, and an equivalent resistor. During experimentation, the voltage across the exploding foil is recorded by the high-voltage probe, while the current is captured via the Rogowski coil. The experimental parameters were set with a capacitance of 0.22 µF and an operational voltage ranging from 900 to 1100 V. While testing the electrical explosion, the eruption of the metal bridge foil after the electrical explosion of the EFI was observed under an optical microscope.

### 3.3. Flyer Velocity Test

The primary function of the EFIs is predicated on converting electrical energy into the kinetic energy that will propel a flyer at high velocities. The trajectory and velocity of this flyer are paramount, as it impacts a primer to initiate a secondary explosive charge. Consequently, the velocity of the flyer emerges as a critical factor when evaluating the efficacy of the EFIs.

**Figure 9:**
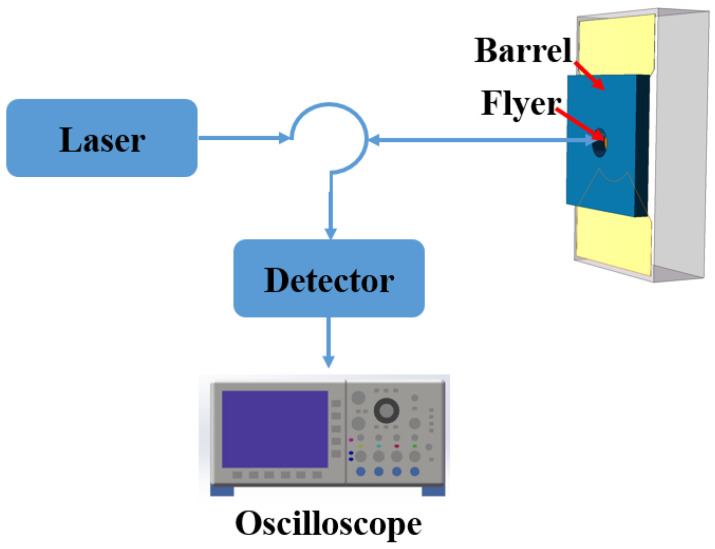
Principle of the flyer velocity test based on PDV.

To investigate the acceleration process of the flyer, photonic Doppler velocimetry (PDV) was employed, which is based on the principle of photon Doppler shift [33]. Figure 9 shows the basic principle of the PDV flyer velocity test. A laser probe dispatches a reference beam to the barrel, where it interacts with light reflected from the moving flyer. The interference of these light sources generates a Doppler beat signal. This optical interference pattern is then converted into an electrical signal by a photoelectric converter. The resultant signal is captured by an oscilloscope. Through fast Fourier transform analysis, the velocity profile of the flyer is accurately deduced. In this test, bridge-wing-thickened EFI chips were integrated with planar switches and capacitors and were then subjected to flyer velocity testing at 900 V/0.22 μF.

## 4. Results and Discussion

### 4.1. Ablation of EFI Chips after an Electrical Explosion

**Figure 10:**
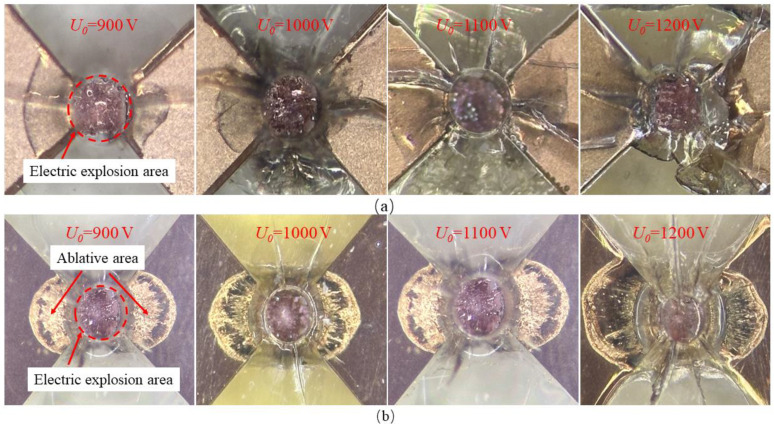
EFI chip ablation after electrical explosions at different operating voltages. (**a**) Bridge-wing thickened bridge foil. (**b**) Conventional square bridge foil.

Figure 10 presents the comparison of the bridge area after the electrical explosion between the bridge-wing-thickened EFI and the conventional square bridge EFI under the conditions of 900 V to 1200 V, with a capacitance of 0.22 μF. It is observed that the electrical explosion in the thickened bridge-wing variant occurred exclusively within the metallic bridge zone, without significant phase changes in the bridge-wing region. Conversely, for the conventional square bridge EFI, its electrical explosion range extended significantly into the bridge-wing area, resulting in severe ablation of the bridge-wing area. The extent of this ablation escalated with rising operating voltages.

The primary reasons for the differing ablation conditions are mainly associated with the current density in the bridge area. Only when the current density exceeds a specific value will it cause the metal to undergo an electrical explosion. The current density is closely related to the resistance of the metal. The resistivity of a metallic conductor, when its length remains unchanged, is determined by its cross-sectional area, exhibiting an inversely proportional relationship. The pre-enhancement thickness of the EFI bridge-wing was measured at 4 μm, increasing to 19 μm post-enhancement. Consequently, the resistance of the pre-enhanced bridge-wing was calculated to be 4.75 times greater than that of the post-enhanced wing. Furthermore, when the overall resistance of the EFI chip was assessed before and after the bridge-wing enhancement, it was observed that the resistance diminished from 22 mΩ to 15 mΩ. This significant reduction in resistance within the bridge-wing region, whilst maintaining a constant resistance at the bridge area, is indicative of improved electrical conductivity, which is advantageous for augmenting the energy utilization efficiency of the system. The bridge-wing thickened EFI chip avoids ablation in the bridge-wing area due to its substantially lower resistance as a result of its thickening. When a high-current pulse moves through the metallic bridge foil, the current density at the edges near the bridge is much lower compared to the bridge. Consequently, the thickened bridge-wing area of the EFI chip does not undergo ablation at the transition zone. In contrast, with the conventional EFI chip, the current density at areas close to the bridge-wings is only marginally lower than at the bridge itself. This slight difference results in a metallic explosion across both the bridge and the connected portions of the bridge-wings when subjected to a pulse current, leading to ablation.

Moreover, the comparison between the observed outcomes from the electrical explosion experiments and the electrothermal computational models in the bridge region reveals a significant congruence. This strongly suggests that the computational model is highly capable of predicting the temperature field distribution with accuracy during the metal electrical explosion process.

### 4.2. Electrical Explosion Performance of Bridge-Wing Thickened EFI

**Figure 11:**
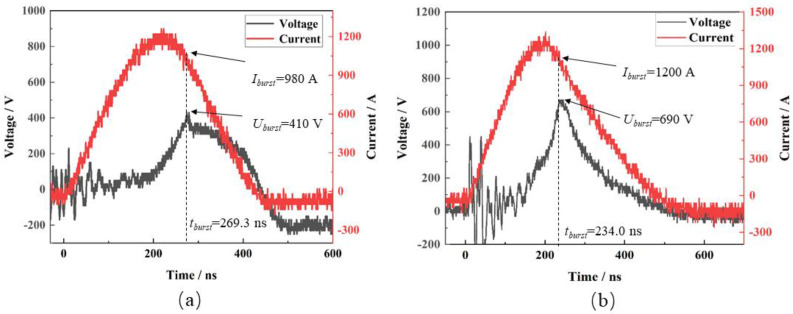
Typical discharging curves of the bridge-wing thickened EFI in conditions of (**a**) 900 V/0.22 μF and (**b**)1000 V/0.22 μF.

Figure 11 shows a typical curve of the burst current and voltage of the electrical explosion, as measured by the test rig. The energy utilization of the EFI was calculated using the following equation:

(9) $$\left\{\begin{array}{l} E_{b}=\int_{0}^{t_{b}}U\left(t\right)\cdot I\left(t\right)dt\\ \eta_{b}=\frac{2E_{b}}{CU_{0}^{2}} \end{array}\right.$$

where *E_b_* is the energy deposited in the bridge area at the burst moment, mJ; this is calculated by integrating the burst voltage, *U*, and the burst current, *I*. *T_b_* is the burst moment, ns; *η_b_* is the energy utilization rate, %; *C* is the capacitance capacity, μF; and *U_0_* is the operating voltage, V.

Table 1 delineates a suite of electrical explosion parameters. An analysis of Table 1 shows that with an ascending operating voltage, there is concurrent augmentation in the burst voltage, burst current, peak current, and energy deposition. Inversely, the burst time, the time of peak current, and the interval between these two metrics exhibit a downtrend with the escalation of operating voltage. This is because the higher the initial voltage, the more energy the capacitor provides to the bridge foil per unit time, and the faster the bridge foil resistance grows, leading to an earlier burst time and an increase in the burst voltage, burst current, and deposition energy. Notably, energy efficiency does not exhibit relentless growth in tandem with operational voltage enhancements. It is observed that upon escalating from an operational voltage of 900 V to 1000 V, the energy efficiency surges from 24.6% to 33.0%. Nevertheless, further elevation to 1100 V causes a regression in energy efficiency to 31.6%. This indicates that there is an optimum matching range between the energy utilization of the exploded foil and the operating voltage. This is because a bypass discharge phenomenon occurs on the surface of the bridge foil that varies with the initial operating voltage, which causes the capacitive energy to not be effectively deposited in the bridge foil, thus affecting the energy utilization of the EFI.

### 4.3. Velocity of the Flyer as Measured by PDV

**Figure 12:**
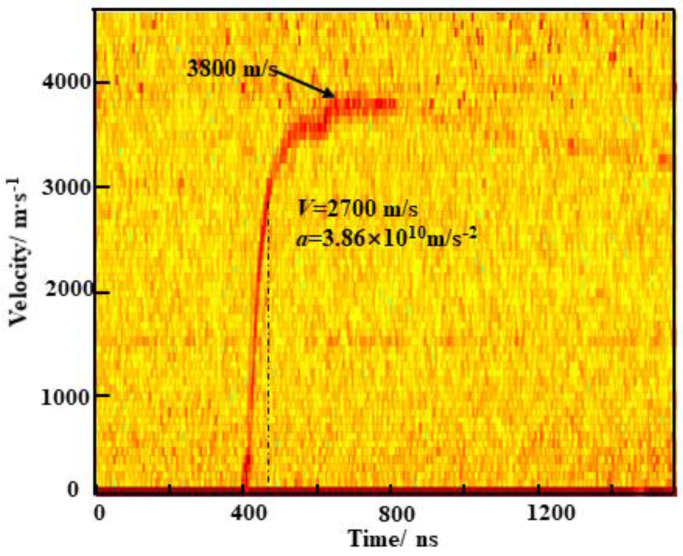
Velocity of the bridge-wing thickened EFI at a condition of 900 V/0.22 μF.

Figure 12 shows the measured velocity of the flyer of the EFIs under the condition of 900 V/0.22 μF. As can be seen from the velocity curve, there are two stages in the acceleration process. Initially, the flyer is propelled by a plasma burst, inducing rapid acceleration of up to 3.86 × 10^10^ m/s^2^, allowing the flyer to achieve a velocity of 2700 m/s within 70 ns. The pressure generated by the plasma at this time is much greater than the flyer and the acceleration of the barrel wall, as well as the resistance of the air. As the pressure of the plasma gradually decreases, the acceleration continues to decrease until, finally, the flyer reaches a maximum velocity of 3800 m/s after 130 ns in the second stage.

## 5. Conclusions

In this study, a bridge-wing thickened type of EFI chip was designed and fabricated using MEMS technology. The results showed that the phenomenon of bridge-wing ablation during the electrical explosion process can be significantly diminished by thickening the bridge-wing. This design notably enhances the electrical energy’s concentration in the bridge area for energy deposition, thereby heightening the energy efficacy of EFIs. The simulation calculation results highly overlap with the experimental results, indicating that the established metal electrical explosion temperature field calculation model can effectively predict the metal electrical explosion process. The current computational model for the metallic electrical explosion process is primarily empirical, being predicated on the occurrence of spark ablation within the bridge-wing region. This assumption, however, is misaligned with the characteristics of EFIs featuring thickened-bridge-wings. Consequently, in the future, the electrical explosion model will be refined to accurately reflect scenarios involving low-energy EFI, where bridge-wing ablation is not present.

## Figures and Tables

**Table 1:** Electrical explosion properties of the bridge-wing thickened EFI.

Capacitance/μF	Operating Voltage/V	Burst Time/ns	Burst Voltage/V	Burst Current/A	Peak Current/A	Peak Current Time/ns	Burst Energy/mJ	Energy Utilization/%
0.22	900	269.3	410	980	1250	218.5	21.9	24.6
	1000	234.0	690	1200	1315	199.1	36.3	33.0
	1100	226.7	720	1360	1568	190.1	42.0	31.6

## Data Availability

All data are contained in the article.
